# Blood pressure management in acute intracerebral haemorrhage: a Time–Trajectory–Phenotype framework

**DOI:** 10.3389/fneur.2026.1900479

**Published:** 2026-10-29

**Authors:** Xin Ping Xia, Yu Zhang, Ling Zhao

**Affiliations:** 1 Department of Rehabilitation, The People's Hospital Medical Group of Xiangzhou, Zhuhai, Guangdong, China; 2 Department of Intensive Care Unit, The People's Hospital Medical Group of Xiangzhou, Zhuhai, Guangdong, China

**Keywords:** blood pressure management, blood pressure variability, haematoma expansion, intracerebral haemorrhage, patient phenotype, time-in-target range, ultra-early intervention

## Abstract

Blood pressure (BP) lowering is a key component of acute intracerebral haemorrhage (ICH) care. Its clinical benefit may depend on how early an appropriate BP range is reached, how steadily it is maintained, and how well the patient tolerates the reduction. We propose a Time–Trajectory–Phenotype framework to examine these dimensions together. Our central hypothesis is that early attainment and stable maintenance of an appropriate BP range may offer greater net benefit than delayed or fluctuating control when further haematoma expansion remains preventable and BP reduction is tolerated. Time encompasses treatment initiation and target attainment. Trajectory captures reduction magnitude and rate, subsequent variability, and sustained exposure within and outside the intended range. Phenotype distinguishes baseline expansion risk from cerebral and systemic tolerance of BP reduction. Randomised trials, serial imaging, and achieved-exposure analyses provide the clinical rationale for examining these dimensions together. The first three hours provide a clinically motivated focus for investigating a therapeutic opportunity that changes over time and varies between patients. Clinically, the framework supports early, sustained guideline-based control and avoidance of excessive reduction within coordinated ICH care. BP variability and time-in-target range are proposed as candidate metrics for prospective comparisons of treatment delivery. These studies should test whether faster target attainment and more stable control improve functional outcomes and safety, and whether treatment effects vary across patient phenotypes.

## 1 Introduction

Spontaneous intracerebral haemorrhage (ICH) remains a devastating stroke subtype associated with high mortality and long-term disability. Following the initial vascular rupture, continuing bleeding can enlarge the haematoma while secondary tissue injury begins to evolve, creating a time-sensitive opportunity for intervention. Given that blood pressure (BP) can be closely monitored and rapidly modified, elevated BP represents a readily accessible target for acute management. The challenge is to reduce arterial pressure that may sustain ongoing bleeding while preserving adequate cerebral and systemic perfusion (1, 2).

Landmark clinical trials have yielded differing results regarding the benefits and risks of intensive BP reduction, showing that a lower absolute target does not necessarily improve functional recovery (3, 4). While guidelines recommend early, smooth, and sustained BP control for eligible patients (5, 6), how the dynamic quality of the BP-lowering process relates to clinical outcomes remains insufficiently elucidated. A single BP measurement can mask very different haemodynamic trajectories: one patient may achieve the target rapidly and remain stable, whereas another may experience prolonged hypertension, substantial fluctuations, or hypotensive episodes. Furthermore, an identical BP-lowering trajectory may carry different risk–benefit profiles depending on baseline vulnerabilities in cerebral and renal perfusion.

To address these disparities, we propose the Time–Trajectory–Phenotype framework. Time encompasses both the onset-to-treatment delay and the interval from treatment initiation to actual target achievement. Trajectory accounts for the magnitude and rate of the initial reduction, subsequent BP variability, and the duration of deviations outside the target range. Phenotype denotes baseline patient characteristics influencing haematoma expansion risk and tolerance of BP reduction. Our central hypothesis is that, when further haematoma expansion remains potentially preventable and physiological reserve permits, achieving an appropriate BP range early, followed by sustained maintenance and avoidance of excessive reduction, offers greater net clinical benefit than delayed or unstable BP control.

We first synthesise human clinical evidence and then examine the biological mechanisms that could explain the effects of treatment timing and BP stability. The first 3 hours provide a focused interval for studying how time, trajectory, and phenotype interact during active haematoma growth. Finally, we examine gut–brain interactions in post-ICH inflammation and the potential role of autonomic modulation in stabilising BP.

## 2 Clinical evidence and controversies

### 2.1 Target intensity and safety

In exploring BP-lowering strategies for acute ICH, the landmark INTERACT2 and ATACH-II trials have informed the balance between treatment intensity, benefit, and risk. INTERACT2 enrolled 2,839 patients within 6 h of onset and compared intensive BP lowering (systolic BP (SBP) < 140 mmHg within 1 h) with guideline-recommended treatment (<180 mmHg). Intensive treatment did not significantly reduce the primary binary endpoint of death or major disability (modified Rankin Scale [mRS] 3–6) at 90 days (odds ratio [OR] 0.87, 95% confidence interval [CI] 0.75–1.01; *p* = 0.06).

However, the prespecified ordinal analysis showed a modest but statistically significant favourable shift in mRS scores (common OR for greater disability 0.87, 95% CI 0.77–1.00; *p* = 0.04). These findings suggest a potential functional benefit, although the primary binary endpoint remained nonsignificant (3).

ATACH-II enrolled 1,000 patients within 4.5 h of onset and compared a more intensive systolic target of 110–139 mmHg with a standard target of 140–179 mmHg using a nicardipine-based intravenous regimen. The more intensive strategy did not improve 3-month functional outcomes compared with standard treatment and was associated with a significantly higher incidence of renal adverse events within 7 days (9.0% vs. 4.0%; *p* = 0.002). This finding reinforces the need to consider systemic organ safety alongside the intended cerebral benefits of BP reduction (4).

Although these trials differ in inclusion criteria, baseline BP, pharmacological protocols, and outcome definitions, they highlight the need to evaluate more than a single target value. Neither trial prospectively randomised patients to compare early versus late target achievement or smooth versus fluctuating trajectories under the same target (3, 4). These findings raise the possibility that treatment timing, the magnitude of BP reduction, and subsequent BP stability contribute to clinical outcomes beyond the absolute target alone. From a physiological perspective, delayed treatment may reduce the opportunity to prevent further haematoma expansion, whereas excessive reductions may compromise perfusion in patients with limited cerebral or renal reserve. These considerations provide a rationale for investigating the timing of initiation, the magnitude and rate of reduction, and the quality of target maintenance alongside the absolute BP target (7).

### 2.2 Earlier treatment and diagnosis

Moving treatment earlier, INTERACT4 investigated the safety and efficacy of prehospital, ambulance-delivered BP reduction within 2 h of symptom onset in patients with suspected stroke, targeting systolic BP of 130–140 mmHg. While the functional outcome for the overall cohort was neutral, stroke-type subgroup analyses showed contrasting clinical effects: patients subsequently diagnosed with haemorrhagic stroke, predominantly ICH, had better functional outcomes, whereas those with cerebral ischaemia had worse outcomes. These findings support the potential benefit of ultra-early BP lowering in ICH, but do not isolate the effects of earlier initiation from those of treatment intensity. The opposing effects also underscore the importance of diagnostic certainty: expedited ICH-specific protocols should be based on rapid, reliable neuroimaging differentiation (8).

Clinical evidence also highlights a gap between imaging outcomes and functional recovery. An individual-participant-data (IPD) meta-analysis encompassing 16 trials and 6,221 patients found that BP-lowering interventions reduced haematoma expansion without an overall improvement in functional outcomes. The analysis covered a broad acute-phase population, with a median onset-to-randomisation time of 3.8 h, and identified heterogeneity in functional effects by strategy and agent. The dissociation between expansion and recovery makes both functional outcome and systemic safety essential endpoints alongside serial imaging when evaluating BP delivery strategies (9).

Table 1 summarises the clinical evidence underpinning the framework. Randomised studies test assigned protocols; analyses of achieved timing and BP trajectories describe exposures that also reflect disease severity and treatment response.

**Table 1:** Clinical evidence for acute BP management.

Study and design	Population and exposure	Principal finding and relevance
INTERACT2 Randomised (3)	2,839; ≤6 h; SBP < 140 versus <180 mmHg.	Primary binary outcome *p* = 0.06; ordinal analysis favoured intensive treatment. Supports a possible modest functional benefit.
ATACH-II Randomised (4)	1,000; ≤4.5 h; SBP 110–139 versus 140–179 mmHg.	No primary functional benefit; more renal adverse events with the lower target. Intensity and systemic safety require joint assessment.
INTERACT4 Randomised (8)	2,404 with suspected stroke; ≤2 h; ambulance-delivered treatment.	Overall neutral; haemorrhagic-stroke subgroup favoured treatment, cerebral-ischaemia subgroup showed harm. Diagnosis and treatment intensity affect interpretation.
IPD meta-analysis (9)	6,221 from 16 trials; median randomisation 3.8 h.	Less expansion without overall functional improvement. Imaging benefit alone does not establish clinical benefit.
Pooled INTERACT Secondary analysis (13)	11,312 from four trials; CT substudy 2,921.	Functional benefit in the full cohort; CT-substudy time modelling suggested diminishing effects. Does not establish an abrupt three-hour cutoff; includes bundled care.
RAINS Observational (15)	312; rapid, intensive, sustained BP protocol.	Earlier target achievement associated with less expansion. Achieved timing was not randomised.
INTERACT3 Cluster randomised (66)	7,036; BP, glucose, temperature, and anticoagulation care bundle.	Improved six-month functional-outcome distribution. Supports coordinated care; does not isolate the BP component.
Time-to-target analysis (14)	5,761 trial participants; timing of sustained SBP 120–140 mmHg assessed.	Earlier sustained control associated with better function and less expansion. Achieved timing was not randomised.
ICHADAPT-2 Randomised (36)	162 randomised; MRI outcome available in 79.	No detected increase in DWI ischaemic lesions with intensive treatment. Precision and incomplete MRI ascertainment limit safety inference.

BP, blood pressure; CT, computed tomography; DWI, diffusion-weighted imaging; IPD, individual-participant data; SBP, systolic blood pressure. Time windows indicate trial eligibility or observed onset-to-randomisation time.

## 3 Timing and therapeutic opportunity

### 3.1 Time-dependent haematoma growth

Serial imaging studies show that haematoma growth is often fastest early after onset and slows thereafter, although its course varies substantially between patients (10–12). This concentration of expansion risk makes ultra-early intervention particularly relevant to preventing additional injury. Time alone, however, cannot identify how much further growth remains possible: some haematomas stabilise early, whereas others continue to expand. Evaluating BP-lowering strategies therefore requires relating treatment timing to the patient’s baseline risk of further expansion. Figure 1 illustrates the heterogeneity of haematoma growth trajectories, including early stabilisation and continued expansion beyond 3 hours.

**Figure 1:**
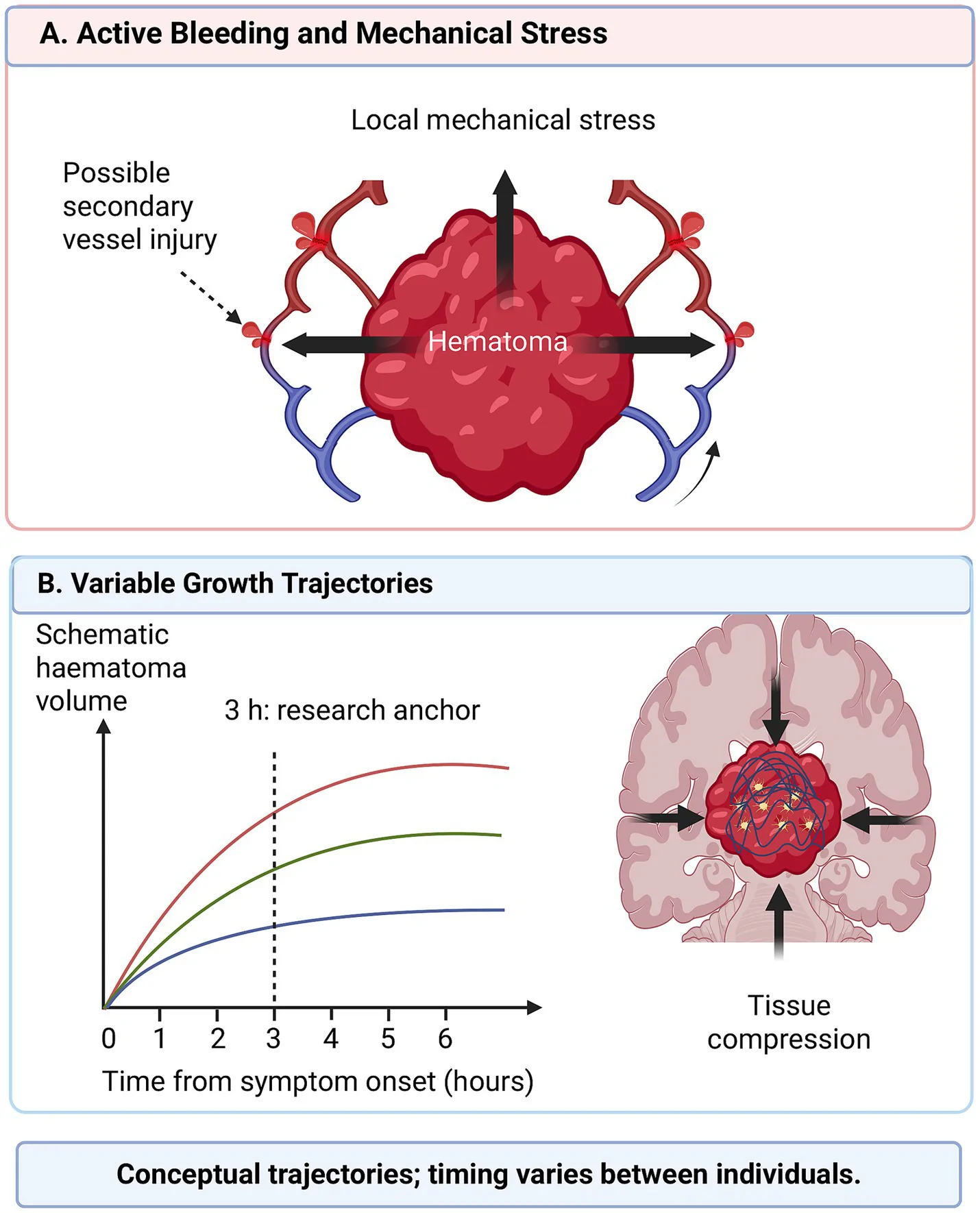
Haematoma expansion over time. **(A)** Local mechanical stress during active bleeding, with possible secondary vessel injury shown as a hypothesised contributor to expansion. **(B)** Schematic trajectories illustrate early stabilisation and continued growth beyond 3 hours. Tissue compression depicts the local mechanical environment. The three-hour line is a research reference; growth and stabilisation vary between individuals. Curves are conceptual illustrations, not fitted patient data. Created in BioRender. Zhang, Y. (2026) https://BioRender.com/ixwwplz.

The timing of baseline CT defines the portion of haematoma evolution captured as subsequent expansion. Growth occurring before the first scan is incorporated into the baseline volume; earlier imaging allows more of the early growth phase to be assessed through follow-up CT (11). The interval between scans also shapes interpretation: dividing the volume change by the elapsed time yields an average growth rate across that period, without identifying when growth accelerated or stopped. Both onset-to-baseline and baseline-to-follow-up intervals should therefore be considered alongside treatment timing when comparing imaging outcomes. Their alignment helps determine which observed growth occurred before treatment and which remained available for an intervention to influence.

### 3.2 Why the first three hours

The 2025 pooled analysis of four INTERACT trials reported improved functional outcomes with intensive treatment in the full cohort. In its CT substudy of 2,921 patients, the overall effects on relative and absolute haematoma growth were not statistically significant. Treatment-by-time modelling within this substudy nevertheless suggested diminishing effects on function and relative growth with later randomisation, with point estimates crossing the null effect line at approximately 3 hours (13). These findings provide a clinical rationale for concentrating research on the first 3 hours, when the opportunity to influence further growth may be greatest.

This pattern is consistent with a narrowing opportunity to prevent additional injury as active bleeding subsides. The duration of that opportunity should vary with bleeding dynamics, haemostatic status, and treatment tolerance. The modelled three-hour crossover is a statistical estimate, not a measurement of haemostasis, and the pooled trials differ in delivery and co-interventions. Three hours therefore serves as a research reference within a continuous time-dependent process. Patients presenting later still require treatment according to clinical indications. Studies should record uncertain onset and prespecify its handling separately from known-onset cohorts.

### 3.3 Treatment initiation and target achievement

Onset-to-drug time reflects access to treatment and delivery logistics; onset-to-target time additionally captures drug administration, dose titration, and the patient’s physiological response. In the Blood Pressure in Acute Stroke Collaboration analysis, earlier control among participants who attained sustained SBP of 120–140 mmHg was associated with better functional outcomes and less haematoma expansion (14). In RAINS, a prospective observational cohort of 312 patients treated with a rapid, intensive, and sustained protocol, earlier target attainment was associated with less haematoma expansion (15). These observations make achieved-target time an important metric, although patients who respond rapidly may differ in baseline severity and treatment tolerance from those who respond more slowly.

Future prospective studies should separately record symptom onset, diagnostic imaging, initial drug administration, first target attainment, and the duration of sustained maintenance. A single in-range reading captures target attainment but does not establish sustained control. Randomised comparisons of delivery protocols designed to shorten time-to-target and improve target maintenance, while retaining the same BP range and safety criteria, would test these strategies more directly than retrospective comparisons based on achieved pressure. Such designs would also reduce the bias introduced by selecting patients according to their response to treatment.

## 4 Blood pressure trajectory

### 4.1 Variability and excessive reduction

The trajectory hypothesis concerns the continuous haemodynamic exposure after treatment begins. Mean blood pressure alone can obscure clinically relevant excursions: a promptly achieved target may be followed by repeated rebounds above range, while a low average may conceal clinically important hypotensive episodes. Post-hoc INTERACT2 and ATACH-II analyses consistently associate greater systolic BP variability (BPV) with poorer outcomes (16, 17). While these associations justify measuring fluctuations, interpretation should consider potential reverse causation—greater BPV may partly reflect autonomic disturbance secondary to severe injury, rather than merely suboptimal care.

Trajectory analyses extend this concept from individual fluctuations to the overall longitudinal shape of the pressure course. An ATACH-II analysis and a 2026 pooled analysis of 11,269 patients linked temporal patterns of achieved SBP to outcomes; the pooled analysis identified six trajectory groups (18, 19). Group membership in these studies, however, was derived observationally rather than assigned randomly. These observed response patterns can inform delivery strategies for prospective testing.

Furthermore, the magnitude of the initial blood pressure fall adds prognostic information that the final stabilised value cannot provide. Pooled INTERACT2/ATACH-II data showed a J-shaped association between reduction magnitude and outcome, and a later ATACH-II analysis linked this early reduction to expansion and function (20, 21). Similarly, an emergency-department study of 420 patients associated “overshooting” below 120 mmHg with worse discharge outcomes (22). Together, these studies support recording the starting value, pressure nadir, reduction magnitude, and duration below the intended range. The clinical significance of a given fall depends on its starting pressure and the patient’s treatment tolerance.

### 4.2 Time in target range

Time-in-target range (TTR) describes the proportion of evaluable time during which SBP remains within a prespecified interval. Cardiovascular literature provides a precedent for this approach, and ICH target-adherence studies demonstrate the importance of whether the intended treatment is actually delivered (23, 24). We propose TTR as a candidate measure of sustained control. By quantifying the duration of in-range exposure, it distinguishes sustained control from a single in-range reading and complements the fluctuation described by BPV and the pressure level described by mean SBP.

$$\begin{array}{c} \mathrm{TTR}\;()=[\text{time within the prespecified range}/\\ \text{total evaluable monitoring time}]\times 100 \end{array}$$

Interpretation requires the target interval, time origin, and observation window to be specified before analysis. The choice of time window defines the clinical question: the first 60 min after drug initiation evaluates pharmacological delivery, whereas a window covering the first 3 hours after onset evaluates how this delivery intersects with the disease course. Monitoring that starts after symptom onset cannot supply measurements for the preceding interval. The proportion of the onset-based window actually observed should be reported. A time-weighted calculation between valid readings may be used if interpolation rules and maximum allowable gaps are prespecified. When time weighting is unavailable, the proportion of readings in range should be reported simply as such.

TTR should be accompanied by above-range and below-range exposure, mean SBP, and a prespecified BPV measure. Two patients with the same TTR may spend their remaining time on opposite sides of the target range, with different implications for efficacy and safety. BPV estimates depend on the recording schedule and metric used; published studies differ in both respects (25, 26). One practical research approach is automated five-minute readings during active titration, supplemented by higher-frequency data where available, with feasibility, coverage, and missingness reported. Ultimately, validation must ask whether TTR adds prognostic information beyond time-to-target, mean pressure, and BPV, and whether prospectively improving it actually changes clinical outcomes.

## 5 Phenotype and treatment tolerance

### 5.1 Identifying expansion risk

The phenotype framework addresses two distinct clinical dimensions: the potential magnitude of additional injury that treatment might prevent (expansion risk), and the patient’s physiological capacity to withstand the haemodynamic intervention (treatment tolerance).

To characterise expansion risk, baseline haematoma volume, intraventricular extension, anticoagulant exposure, and imaging heterogeneity are relevant factors. Non-contrast CT (NCCT) markers, notably the blend and black-hole signs, have been associated with expansion in validation studies and diagnostic meta-analyses (27–29). The GIVE score combines Glasgow Coma Scale, the island sign, intraventricular haemorrhage, and onset-to-NCCT time; its performance and calibration should be reassessed before transfer to new clinical populations (30).

Higher baseline risk can yield a larger absolute benefit even when the relative treatment effect remains constant. This concept of “prognostic enrichment” should be distinguished from identifying a true predictive biomarker that modifies the relative efficacy of the intervention or justifies a tailored BP target. For instance, a prospective analysis found no detectable interaction between the CT angiography (CTA) spot sign and rapid target achievement regarding haematoma expansion or functional outcome (31). NCCT heterogeneity and CTA contrast extravasation also capture different imaging features of expansion risk. Using such markers to customise treatment intensity requires evidence from adequately powered treatment-by-marker interaction analyses.

### 5.2 Vulnerability and physiological reserve

Treatment tolerance depends on cerebral perfusion dynamics, systemic physiological reserve, and the acute mass effect of the existing haemorrhage. Haemorrhage volume and anatomical location primarily drive direct neurological injury, whereas intraventricular bleeding, hydrocephalus, and significant mass effect can create competing urgent haemodynamic and neurosurgical priorities. Because cerebral perfusion pressure (CPP) is the difference between mean arterial pressure and intracranial pressure (ICP), aggressive arterial BP reduction in the setting of severely elevated ICP can compromise the pressure gradient essential for brain perfusion. Therefore, the presenting blood pressure should be interpreted—alongside clinical severity, prior hypertension, medication history, and renal function—rather than being viewed merely as a direct surrogate for active bleeding. Clinical guidelines distinguish the evidence available for mild-to-moderate ICH from the therapeutic uncertainty surrounding large or severe haemorrhages (5).

Underlying vasculopathy provides additional context. Hypertensive arteriopathy and cerebral amyloid angiopathy (CAA) have different pathological distributions, and hypertension-associated small-vessel disease frequently coexists with diffuse vascular injury (32, 33). The Boston criteria version 2.0 provide MRI-based criteria for identifying probable CAA when clinically appropriate (34). Aetiological classification informs subsequent risk stratification, whereas immediate treatment decisions depend on the current bleeding and perfusion risks.

In the observational Ethnic/Racial Variations of Intracerebral Haemorrhage (ERICH) MRI cohort, the association between SBP reduction from presentation to 24 h after admission and diffusion-weighted imaging (DWI) lesions differed according to whether patients had hypertension and were receiving antihypertensive treatment before ICH (35). Among the 79 participants with evaluable MRI in the randomised ICHADAPT-2 trial, DWI lesions occurred in 13 of 42 patients assigned to an SBP target below 140 mmHg and 14 of 37 assigned to a target below 180 mmHg; lesion frequency and volume did not differ significantly between groups (36). Within the Time–Trajectory–Phenotype framework, prior hypertension and its treatment history are considered alongside haemorrhage severity and current perfusion risks when assessing tolerance of BP reduction.

Finally, a 2026 stepwise meta-analysis of ATACH-II, INTERACT2, and INTERACT3 did not demonstrate a statistically significant functional benefit from intensive treatment in either deep or lobar ICH (37). This analysis combined trials with different outcome definitions and included a care-bundle intervention. Anatomical location alone therefore does not establish a differential BP prescription. In this proposed framework, phenotype represents a multidimensional baseline assessment used to evaluate absolute benefit, relative treatment efficacy, and physiological safety. Separating these dimensions clarifies which features predict prognosis, which might modify treatment effects, and which require closer safety monitoring.

### 5.3 Origins of BP elevation

BP elevation after ICH can arise from several interacting processes: pre-existing hypertension, sympathetic activation after disruption of central autonomic regulation, pain or agitation, and a compensatory response to raised ICP. Haematoma size, location, and intraventricular extension may influence these responses through injury severity and pressure effects. This makes the acute BP surge both a potentially modifiable exposure and a possible consequence of the haemorrhage (38, 39).

Peripheral feedback also contributes to pressure stability. In mice, combined deletion of Piezo1 and Piezo2 in baroreceptor sensory ganglia abolished the baroreflex and produced labile hypertension with increased BPV (40). This identifies both channels as components of neuronal pressure sensing. The evidence derives from baroreceptor models; ICH-specific changes in this pathway remain uncharacterised. This pressure-regulatory function is distinct from the endothelial channels and protease pathways involved in barrier injury or oedema.

## 6 Biological rationale

Two overlapping biological processes could explain why treatment timing and the course of BP matter beyond the achieved pressure. During active bleeding, haemodynamic load may influence the mechanical stability of local haemostasis. Extravasated blood also initiates barrier disruption, inflammation, and altered fluid transport, which evolve over hours to days (41, 42). Early pressure control therefore intersects with both a forming haemostatic plug and an evolving tissue response, providing distinct mechanisms through which treatment timing and BP stability could influence outcome.

### 6.1 Pressure and local haemostasis

Experimental fibrin studies link clot strength to its microarchitecture, cross-linking density, and the mode of mechanical loading. Factor XIIIa-mediated cross-linking contributes to rupture resistance, while shear-loading experiments show that failure behaviour depends on both applied load and internal structure (43, 44). Investigations of fibrin-network elasticity and fracture mechanics further demonstrate that cross-linking alone does not determine structural integrity (45, 46). Together, these material findings connect clot architecture with load tolerance, providing a basis for investigating how pressure exposure interacts with a forming haemostatic plug.

Clinical observations provide a complementary haemostatic signal. In a single-centre registry-based cohort of 132 patients, lower admission FXIII antigen levels were associated with haematoma expansion and adverse outcomes (47). FXIII was measured as admission antigen rather than serial functional activity. The association therefore concerns haemostatic vulnerability, while the evolution of FXIII-dependent clot stability after ICH remains unresolved.

Repeated deformation can contribute to fibrin-clot damage and failure in cyclic-loading and molecular-creep experiments (48, 49). These findings motivate the hypothesis that recurrent pressure excursions could destabilise local haemostasis more than a stable exposure with a comparable mean pressure. At a ruptured vessel, the load on the clot depends on vascular geometry, surrounding tissue pressure, and the transmission of systemic arterial pressure to the clot interface. BPV provides an indirect measure of this exposure; experiments that vary pressure fluctuation while maintaining the same mean pressure could test its contribution to continued bleeding.

Early, stable BP control could favour local haemostasis by reducing recurrent mechanical loading on the forming haemostatic plug. The proposed benefit depends on achieving control while further bleeding remains possible and limiting subsequent pressure excursions. Figure 2 illustrates the proposed relationship between BP trajectory and plug integrity.

**Figure 2:**
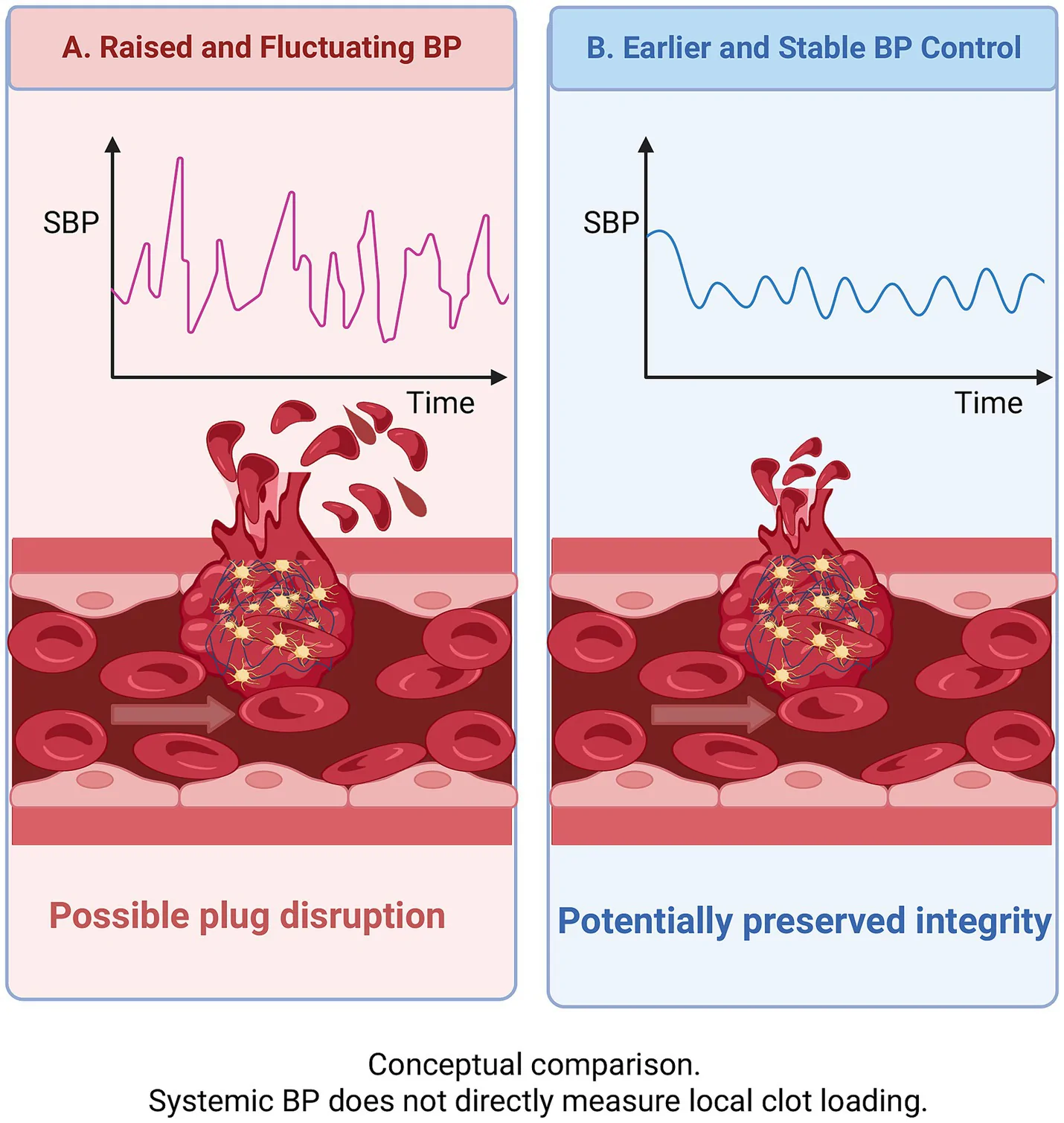
Haemodynamics and local haemostasis. **(A)** Raised and fluctuating BP. **(B)** Earlier and stable BP control. The paired panels illustrate the hypothesised relationship between systemic BP trajectories and the mechanical integrity of a vessel-wall haemostatic plug. The scenarios combine differences in mean pressure, variability, and timing. Systemic BP is an indirect proxy for local clot loading; direct testing requires measurement of pressure transmission and plug stability. Created in BioRender. Zhang, Y. (2026) https://BioRender.com/8d2rque.

### 6.2 Mechanotransduction and barrier injury

Mechanical stress can also activate intracellular signalling. PIEZO1 is a candidate mechanosensor linking tissue deformation or vascular shear to cellular responses, although its proposed role in ICH remains incompletely characterised (50). More direct ICH evidence comes from transient receptor potential vanilloid 4 (TRPV4): in a rat model, pharmacological blockade reduced stress-fibre-associated blood–brain barrier (BBB) disruption and altered the protein kinase C alpha (PKCα)/RhoA/myosin light chain 2 (MLC2) signalling pathway. The principal assessments were conducted at 24–72 h, and MRI showed no significant reduction in haematoma volume (51). This experiment supports a channel–cytoskeleton–barrier mechanism in experimental ICH, while leaving its dependence on BP and its relevance to hyperacute bleeding to be tested.

Sulfonylurea receptor 1–transient receptor potential melastatin 4 (SUR1–TRPM4) provides a separate candidate pathway for post-ICH oedema. A rat study associated glibenclamide treatment with preserved BBB integrity, improved aquaporin-4 (AQP4) polarisation, and reduced oedema (52). In the phase 2 GATE-ICH trial, oral glibenclamide did not significantly improve the primary binary functional endpoint at 90 days. Secondary analyses favoured the mRS distribution and some perihaematomal oedema measures, while asymptomatic hypoglycaemia was more frequent (53). These findings support investigation of post-ICH oedema while leaving channel-specific mediation and its relation to BP control unresolved.

Protease pathways offer another route to barrier injury. In rat ICH, an apolipoprotein E receptor-mimetic intervention linked low-density lipoprotein receptor-related protein 1 (LRP1) activation and cyclophilin A (CypA)/matrix metalloproteinase-9 (MMP-9) inhibition to improved junction-protein expression and reduced permeability; the wider MMP literature describes effects that vary with substrate, model, and phase (54, 55). A separate rat study linked neutrophil extracellular traps (NETs), extracellular signal-regulated kinase (ERK)/MMP-9 signalling, and AQP4 changes to perihaematomal oedema over the first days (56). Barrier injury thus involves interacting inflammatory and proteolytic processes whose effects vary over time.

Calpains are intracellular calcium-regulated proteases. In cultured brain endothelial cells and experimental traumatic brain injury, calpain inhibition preserved junctional organisation and reduced barrier permeability (57). Calpain provides a potential link between calcium-dependent signalling and junctional injury, although its role downstream of BP-driven PIEZO1 activation has not been established in ICH. “Enzymatic overload” describes a proposed imbalance in which proteolytic activity exceeds endogenous inhibition and barrier repair. No quantified temporal threshold has been determined for this imbalance.

Early, stable BP control may limit recurrent mechanical stimulation of the injured microvasculature, providing a potential link between the BP trajectory and the progression of barrier injury. The biological opportunity lies in modifying mechanical exposure as secondary injury evolves. Serial measurements of pressure, cerebral perfusion, and permeability in ICH models, with mean pressure matched across trajectories and direct drug effects controlled, would test this proposed mechanism.

### 6.3 AQP4 and delayed clearance

Brain fluid homeostasis and solute clearance involve multiple routes, including perivascular transport associated with AQP4 (58). In a collagenase-induced mouse ICH model, pharmacological upregulation of AQP4 improved glymphatic measures and reduced haematoma volume at day three, but was also accompanied by larger MRI-defined oedema volumes (59). This dissociation highlights the distinct effects of AQP4-associated transport on blood-product clearance and water accumulation. AQP4 abundance, astrocytic endfoot localisation, and tracer movement remain distinct measurements of the pathway.

Haematoma resorption and the clearance of blood degradation products continue beyond the period of active bleeding (60, 61). Preventing further bleeding and promoting the clearance of existing blood address different stages of recovery, providing complementary opportunities for treatment. A complementary hypothesis is that the early haemodynamic course influences subsequent transport or clearance. Testing it requires relating hyperacute BP and cerebral perfusion measurements to later AQP4 localisation, tracer movement, and blood-product clearance. Figure 3 summarises selected candidate pathways.

**Figure 3:**
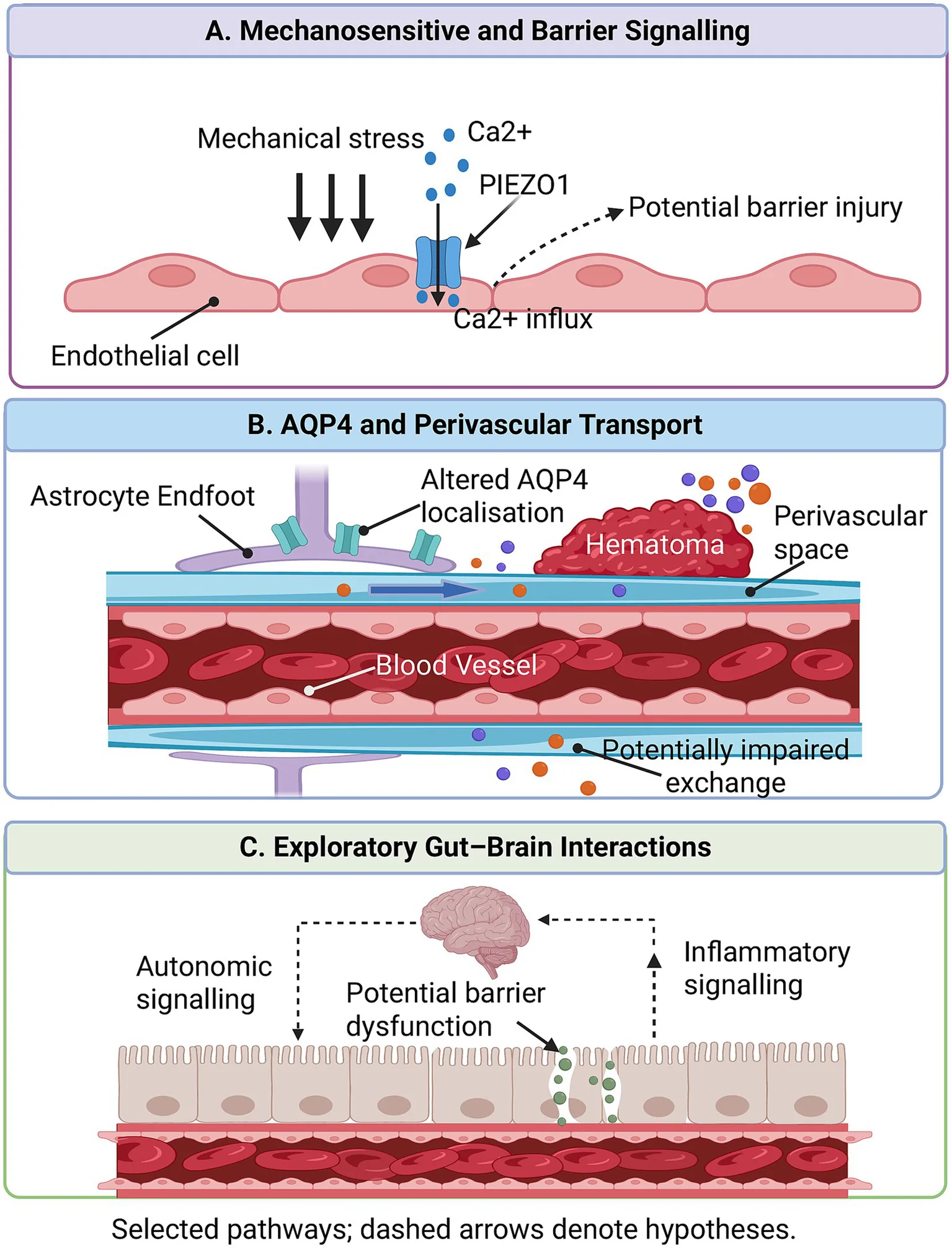
Selected candidate pathways after ICH. **(A)** PIEZO1-mediated calcium entry is depicted as a candidate link between mechanical stress and barrier injury. **(B)** AQP4 localisation and perivascular transport relate to experimental studies of later fluid exchange and clearance. Fluid and solute movement is shown outside the vascular lumen. **(C)** Autonomic and inflammatory gut–brain signalling represent exploratory links between central injury and peripheral responses. Dashed arrows denote hypothesised relationships. Created in BioRender. Zhang, Y. (2026) https://BioRender.com/20hl8qi.

## 7 Clinical implementation

The immediate application of this framework is to improve the delivery and measurement of established care. Upon CT confirmation of ICH, clinicians should evaluate presenting blood pressure, neurological severity, anticoagulant exposure, and neurosurgical indications simultaneously. In mild-to-moderate ICH with presenting SBP of 150–220 mmHg, the 2022 American Heart Association/American Stroke Association (AHA/ASA) guideline supports consideration of a target of 140 mmHg and subsequent maintenance at 130–150 mmHg. For eligible patients, initiating treatment within 2 hours of onset and achieving the target within 1 hour of initiation can be beneficial. Lowering SBP below 130 mmHg may be harmful. Large or severe haemorrhages and cases requiring surgical decompression warrant individual assessment because evidence for intensive BP lowering in these groups remains limited (5). European guidelines similarly advocate early reduction in appropriate small-to-moderate ICH while acknowledging uncertainty about patient selection and targets (6, 62).

Achieving a predictable haemodynamic trajectory requires suitable pharmacological agents and a reliable delivery system. Rapid-onset intravenous agents that facilitate minute-to-minute dose adjustment—such as nicardipine or clevidipine infusions—allow titratable control; labetalol or locally available urapidil serve as viable alternatives depending on patient contraindications and institutional protocols. Agent selection should be driven by cardiac and respiratory comorbidities, formulation availability, and nursing familiarity. Comparative evidence has not established a preferred agent for improving functional outcome after ICH. Contemporary guidelines and emergency management reviews place BP titration within the broader assessment of physiological deterioration and competing clinical priorities (7, 39, 63).

During active titration, continuous or high-frequency BP measurements should be coupled with neurological assessments, heart rate monitoring, and assessment of systemic perfusion. Continuous arterial monitoring or frequent non-invasive readings can guide titration according to clinical stability and available resources. Comparative evidence defining the optimal monitoring modality and frequency for ICH remains limited (5). Any new neurological deficit, sudden hypotension, or precipitous pressure drop should trigger an immediate reassessment of the treatment and competing aetiologies. Monitoring creatinine and urine output is essential to evaluate renal safety—particularly in patients with pre-existing chronic kidney disease—since acute kidney injury (AKI) post-ICH can stem from a combination of haemodynamic and non-haemodynamic insults (64).

BP control is one component of coordinated, multidisciplinary ICH care. The ABC hyperacute bundle combined anticoagulant reversal, intensive BP lowering, and expedited access to neurosurgery and critical care; its implementation was associated with significantly lower 30-day case fatality in an observational cohort (65). Similarly, INTERACT3 evaluated a comprehensive care bundle addressing BP, glucose, temperature, and anticoagulation abnormalities via a stepped-wedge cluster-randomised trial, demonstrating an improved distribution of functional outcomes (66). The treatment effect in INTERACT3 applies to the combined intervention. A subsequent secondary analysis comparing young and older adults extends the evidence on population heterogeneity within that care bundle (67).

In practice, time is addressed by reducing diagnostic and treatment delays; trajectory by titration and frequent reassessment to sustain control; and phenotype by balancing expansion risk against treatment tolerance. These tasks proceed alongside anticoagulant reversal, dedicated stroke-unit care, and indicated surgical evaluation. Time-to-target, sustained in-range exposure, and cerebral and renal safety events provide measurable outputs for comparing delivery strategies.

## 8 Prospective tests and exploratory extensions

### 8.1 Testing the proposed framework

The framework generates three predictions for prospective testing. First, shortening onset-to-target time should increase treatment benefit when substantial further haematoma expansion remains possible. Randomised delivery protocols with the same BP target, accompanied by a prespecified continuous treatment-by-time analysis, could test this prediction. Second, improving sustained control should provide benefit beyond that explained by the mean pressure alone. Trials should measure TTR, BPV, and above- and below-range exposure, and confirm which aspects of the trajectory actually differ between strategies. An improvement in these metrics without a clinically meaningful improvement in function, safety, or expansion would limit their value as therapeutic targets.

Third, baseline expansion risk and haemodynamic vulnerability should explain differences in net clinical benefit. Studies should distinguish a larger absolute benefit associated with higher baseline risk from a treatment-by-phenotype interaction, and evaluate safety separately. Imaging biomarkers, prior BP control, haemorrhage severity, and renal function are candidates for prespecified assessment. Interaction analyses require adequate power and independent validation. Phenotype can currently inform estimates of expansion risk and monitoring needs; tailoring the BP target to a marker requires a reproducible treatment interaction.

Functional outcome should remain central, assessed using a prespecified mRS endpoint alongside mortality, neurological deterioration, and renal adverse events. Standardised serial imaging provides a mechanistic measure of expansion, while perfusion or DWI assessments can examine cerebral safety. Analyses of achieved haemodynamic exposure should account for baseline severity, anticoagulant reversal, and time-varying treatment, recognising that adjustment does not confer the causal protection of randomisation. Mechanistic studies should manipulate mean pressure and fluctuation separately and select measurements appropriate to active bleeding, subsequent barrier injury, or delayed clearance. Table 2 links the principal predictions to tests and findings that would constrain them.

**Table 2:** Prospective tests of the proposed framework.

Prediction	Prospective test	Finding that would constrain the hypothesis
Timing and preventable expansion (13, 15)	Randomise delivery protocols with the same BP target; record onset-to-target time and test a continuous treatment-by-time interaction.	No clinically meaningful benefit from earlier control despite adequate timing separation and precision.
Sustained trajectory beyond mean SBP (16, 23)	Compare titration strategies; measure TTR, BPV, mean SBP, and above- and below-range exposure.	Improved trajectory metrics without improved expansion, function, or safety despite adequate separation and precision.
Phenotype and net benefit (28, 31, 35)	Prespecify expansion-risk and tolerance features; assess absolute benefit, treatment interaction, and safety separately.	Prognostic value without a reproducible interaction would leave marker-directed intensification unsupported.
Pressure and local haemostasis (48, 49)	Manipulate mean pressure and fluctuation separately in ICH models; measure local load, continued bleeding, and clot stability.	No effect of altered loading on haemostasis despite confirmed pressure separation.
Pressure and barrier injury (51, 52)	Use serial pressure, perfusion, and permeability measurements with controls for direct drug effects.	Barrier changes attributable to the drug rather than the pressure trajectory would weaken the proposed BP-mediated link.
Early control and delayed transport (59)	Manipulate hyperacute pressure trajectories and assess later AQP4 localisation, tracer transport, and blood-product clearance.	No change in delayed transport or clearance despite adequate exposure separation would weaken the mediation hypothesis.
Gut and autonomic modulation (71, 72)	Establish temporal order; assess standardised adjunct feasibility and safety before controlled efficacy and mechanism studies.	Delayed associations or multi-acupoint effects alone would not establish a hyperacute gut- or ST36-specific mechanism.

### 8.2 Brain and gut interactions

Acute brain injury can be accompanied by autonomic, inflammatory, and gastrointestinal dysregulation. Reviews of gastrointestinal dysfunction and the microbiota–gut–brain axis describe potential systemic links after ICH; experimental studies have also examined interventions affecting this axis (68–70). These observations suggest a link between central injury and peripheral inflammatory responses.

A human study of 70 patients with mild-to-moderate ICH reported associations between expansion classification and microbiota or inflammatory profiles. Baseline CT was obtained within 24 h of onset, and stool samples within 48 h of admission. Its expansion definition also included a fall in Glasgow Coma Scale alongside CT volume criteria (71). These measurements cannot establish the temporal ordering of hyperacute BP elevation and intestinal injury. Prospective work should use serial autonomic, haemodynamic, and intestinal-injury measurements, accounting for infection, medication, and enteral nutrition. Demonstrating that a BP intervention alters these intestinal responses would support their role as a mediator of treatment benefit.

### 8.3 Autonomic modulation and ST36

Targeted autonomic neuromodulation could offer an adjunctive approach to BP instability. A small randomised study of 54 patients with cerebral haemorrhage reported early BP and haematoma findings after a multi-acupoint regimen that included ST36 (72). The study did not isolate an ST36-specific effect or establish an effect on BPV or sympathetic activation. Mouse experiments identified a neuroanatomical basis for an ST36-region electroacupuncture intervention to activate the vagal–adrenal axis, while rodent sepsis studies provide additional evidence about anti-inflammatory responses (73, 74). Initial ICH studies should define the stimulation protocol and assess feasibility, safety, autonomic function, and BP trajectories alongside standard hyperacute care. Heart rate variability would provide an indirect measure of autonomic function.

## 9 Conclusion

Hyperacute BP management following ICH is a dynamic physiological exposure whose clinical effects may depend on when control is achieved, how pressure evolves, and the patient’s capacity to tolerate the intervention. The Time–Trajectory–Phenotype framework brings these dimensions into a common, testable hypothesis. The first 3 hours provide a clinically motivated focus, with treatment time analysed continuously across the acute period. Immediate priorities are prompt diagnosis, early attainment and sustained maintenance of guideline-based targets, avoidance of excessive reduction, and coordinated care. Prospective trials should determine whether faster delivery and more stable control improve function and safety, and whether baseline phenotypes identify different treatment effects. Phase-specific experiments on haemostasis, barrier integrity, and fluid transport can establish which mechanisms connect BP delivery to subsequent injury and recovery.

## Data Availability

The original contributions presented in the study are included in the article/supplementary material, further inquiries can be directed to the corresponding author.

## Glossary

AHA/ASA: American Heart Association/American Stroke Association
AKI: acute kidney injury
AQP4: aquaporin-4
BBB: blood–brain barrier
BP: blood pressure
BPV: blood pressure variability
CAA: cerebral amyloid angiopathy
CI: confidence interval
CPP: cerebral perfusion pressure
CT: computed tomography
CTA: CT angiography
CypA: cyclophilin A
DWI: diffusion-weighted imaging
ERICH: Ethnic/Racial Variations of Intracerebral Haemorrhage
ERK: extracellular signal-regulated kinase
ESO: European Stroke Organisation
ICH: intracerebral haemorrhage
ICP: intracranial pressure
IPD: individual-participant data
LRP1: low-density lipoprotein receptor-related protein 1
MLC2: myosin light chain 2
MMP: matrix metalloproteinase
MRI: magnetic resonance imaging
mRS: modified Rankin Scale
NCCT: non-contrast computed tomography
NETs: neutrophil extracellular traps
OR: odds ratio
PKCα: protein kinase C alpha
SBP: systolic blood pressure
ST36: Zusanli acupoint
SUR1: sulfonylurea receptor 1
TRPM4: transient receptor potential melastatin 4
TRPV4: transient receptor potential vanilloid 4
TTR: time-in-target range
FXIII: factor XIII

## References

[1] Parry-Jones AR Krishnamurthi R Ziai WC Shoamanesh A Wu S Martins SO . World stroke organization (WSO): global intracerebral hemorrhage factsheet 2025. Int J Stroke. (2025) 20:145–50. doi: 10.1177/17474930241307876, 39629687 PMC11786522

[2] Puy L Parry-Jones AR Sandset EC Dowlatshahi D Ziai W Cordonnier C. Intracerebral haemorrhage. Nat Rev Dis Primers. (2023) 9:14. doi: 10.1038/s41572-023-00424-7, 36928219

[3] Anderson CS Heeley E Huang Y Wang J Stapf C Delcourt C . Rapid blood-pressure lowering in patients with acute intracerebral hemorrhage. N Engl J Med. (2013) 368:2355–65. doi: 10.1056/NEJMoa1214609, 23713578

[4] Qureshi AI Palesch YY Barsan WG Hanley DF Hsu CY Martin RL . Intensive blood-pressure lowering in patients with acute cerebral hemorrhage. N Engl J Med. (2016) 375:1033–43. doi: 10.1056/NEJMoa1603460, 27276234 PMC5345109

[5] Greenberg SM Ziai WC Cordonnier C Dowlatshahi D Francis B Goldstein JN . 2022 Guideline for the management of patients with spontaneous intracerebral hemorrhage: a guideline from the American Heart Association/American Stroke Association. Stroke. (2022) 53:e282–361. doi: 10.1161/STR.0000000000000407 35579034

[6] Steiner T Purrucker JC Aguiar de Sousa D Apostolaki-Hansson T Beck J Christensen H . European stroke organisation (ESO) and European Association of Neurosurgical Societies (EANS) guideline on stroke due to spontaneous intracerebral haemorrhage. Eur Stroke J. (2025) 10:1007–86. doi: 10.1177/23969873251340815, 40401775 PMC12098356

[7] Mutimer CA Yassi N Wu TY. Blood pressure Management in Intracerebral Haemorrhage: when, how much, and for how long? Curr Neurol Neurosci Rep. (2024) 24:181–9. doi: 10.1007/s11910-024-01341-2, 38780706 PMC11199276

[8] Li G Lin Y Yang J Anderson CS Chen C Liu F . Intensive ambulance-delivered blood-pressure reduction in Hyperacute stroke. N Engl J Med. (2024) 390:1862–72. doi: 10.1056/NEJMoa2314741, 38752650

[9] Moullaali TJ Wang X Sandset EC Woodhouse LJ Law ZK Arima H . Early lowering of blood pressure after acute intracerebral haemorrhage: a systematic review and meta-analysis of individual patient data. J Neurol Neurosurg Psychiatry. (2022) 93:6–13. doi: 10.1136/jnnp-2021-327195, 34732465 PMC8685661

[10] Al-Ajlan FS Gladstone DJ Song D Thorpe KE Swartz RH Butcher KS . Time course of early hematoma expansion in acute spot-sign positive intracerebral hemorrhage: prespecified analysis of the SPOTLIGHT randomized clinical trial. Stroke. (2023) 54:715–21. doi: 10.1161/STROKEAHA.121.038475, 36756899

[11] Abou Karam G Chen M-C Zeevi D Harms BC Torres-Lopez VM Rivier CA . Time-dependent changes in hematoma expansion rate after Supratentorial intracerebral hemorrhage and its relationship with neurological deterioration and functional outcome. Diagnostics (Basel). (2024) 14:308. doi: 10.3390/diagnostics14030308, 38337824 PMC10855868

[12] Mutimer CA Wu TY Zhao H Churilov L Campbell BCV Cheung A . Ultra-early hematoma expansion is associated with ongoing hematoma growth and poor functional outcome. Stroke. (2025) 56:838–47. doi: 10.1161/STROKEAHA.124.050131, 39882606

[13] Wang X Ren X Li Q Ouyang M Chen C Delcourt C . Effects of blood pressure lowering in relation to time in acute intracerebral haemorrhage: a pooled analysis of the four INTERACT trials. Lancet Neurol. (2025) 24:571–9. doi: 10.1016/S1474-4422(25)00160-7, 40541207

[14] Wang X Yang J Moullaali TJ Sandset EC Woodhouse LJ Law ZK . Influence of time to achieve target systolic blood pressure on outcome after intracerebral hemorrhage: the blood pressure in acute stroke collaboration. Stroke. (2024) 55:849–55. doi: 10.1161/STROKEAHA.123.044358, 38410986

[15] Rodriguez-Luna D Pancorbo O Llull L Silva Y Prats-Sanchez L Muchada M . Effects of achieving rapid, intensive, and sustained blood pressure reduction in intracerebral hemorrhage expansion and functional outcome. Neurology. (2024) 102:e209244. doi: 10.1212/WNL.0000000000209244, 38598746

[16] Manning L Hirakawa Y Arima H Wang X Chalmers J Wang J . Blood pressure variability and outcome after acute intracerebral haemorrhage: a post-hoc analysis of INTERACT2, a randomised controlled trial. Lancet Neurol. (2014) 13:364–73. doi: 10.1016/S1474-4422(14)70018-3, 24530176

[17] Qureshi AI Baskett W Gomes JA Lakhani P Rabinstein AA Rose DZ . The association between hourly systolic blood pressure variability and outcomes in patients with intracerebral hemorrhage is time-dependent: post-hoc analysis of the ATACH-2 trial. Neurocrit Care. (2026) 44:647–55. doi: 10.1007/s12028-025-02376-0, 40993486

[18] Tanaka K Koga M Fukuda-Doi M Qureshi AI Yamamoto H Miwa K . Temporal trajectory of systolic blood pressure and outcomes in acute intracerebral hemorrhage: ATACH-2 trial cohort. Stroke. (2022) 53:1854–62. doi: 10.1161/STROKEAHA.121.037186, 35400202 PMC9126256

[19] Wang X Phan TG Ren X Ma HH Li Q Ouyang M . Systolic blood pressure trajectory and outcomes in acute intracerebral hemorrhage: pooled analysis of the 4 INTERACT and ATACH-II clinical trials. Neurology. (2026) 106:e214671. doi: 10.1212/WNL.0000000000214671, 41855454 PMC13035102

[20] Wang X Di Tanna GL Moullaali TJ Martin RH Shipes VB Robinson TG . J-shape relation of blood pressure reduction and outcome in acute intracerebral hemorrhage: a pooled analysis of INTERACT2 and ATACH-II individual participant data. Int J Stroke. (2022) 17:1129–36. doi: 10.1177/17474930211064076, 34983270

[21] Li Q Lv X Morotti A Qureshi AI Dowlatshahi D Falcone GJ . Optimal magnitude of blood pressure reduction and hematoma growth and functional outcomes in intracerebral hemorrhage. Neurology. (2025) 104:e213412. doi: 10.1212/WNL.0000000000213412, 39913881 PMC11803522

[22] Shi AC Taylor T Huang C-C Singhal AB Goldstein JN Bevers MB . Early intensive blood pressure reduction after intracerebral hemorrhage is associated with worse functional outcome: the risk of overshooting blood pressure goals. Ann Emerg Med. (2026) 87:628–39. doi: 10.1016/j.annemergmed.2025.10.009, 41369631 PMC12757810

[23] Al-Dalakta A Tabaja C Motairek I El Hajjar AH Agarwal N St John J . Blood pressure time in target range and its impact on clinical outcomes. Curr Cardiol Rep. (2024) 26:1145–51. doi: 10.1007/s11886-024-02111-9, 39141252 PMC11461595

[24] Leshko NA Lamore RF Zielke MK Sandsmark DK Schmidt LE. Adherence to established blood pressure targets and associated complications in patients presenting with acute intracerebral hemorrhage. Neurocrit Care. (2023) 39:378–85. doi: 10.1007/s12028-023-01679-4, 36788180

[25] Zompola C Palaiodimou L Voumvourakis K Stefanis L Katsanos AH Sandset EC . Blood pressure variability in acute stroke: a narrative review. J Clin Med. (2024) 13:1981. doi: 10.3390/jcm13071981, 38610746 PMC11012361

[26] Patel P Kim A Shapsay M Jaddu S Seyoum NY Ternovskaia A . Emergency department vital sign variability is associated with hematoma progression in spontaneous intracerebral hemorrhage. J Clin Med. (2025) 14:4404. doi: 10.3390/jcm14134404, 40648777 PMC12249892

[27] Morotti A Boulouis G Dowlatshahi D Li Q Shamy M Al-Shahi Salman R . Intracerebral haemorrhage expansion: definitions, predictors, and prevention. Lancet Neurol. (2023) 22:159–71. doi: 10.1016/S1474-4422(22)00338-6, 36309041

[28] Ducroux C Nehme A Rioux B Panzini M-A Fahed R Gioia LC . NCCT markers of intracerebral hemorrhage expansion using revised criteria: an external validation of their predictive accuracy. AJNR Am J Neuroradiol. (2023) 44:658–64. doi: 10.3174/ajnr.A7871, 37169542 PMC10249705

[29] Arba F Rinaldi C Boulouis G Fainardi E Charidimou A Morotti A. Noncontrast computed tomography markers of cerebral hemorrhage expansion: diagnostic accuracy Meta-analysis. Int J Stroke. (2022) 17:835–47. doi: 10.1177/17474930211061639, 34842473

[30] Yang T-N Lv X-N Wang Z-J Hu X Zhao L-B Cheng J . Predicting the risk of hematoma expansion in acute intracerebral hemorrhage: the GIVE score. BMC Neurol. (2025) 25:21. doi: 10.1186/s12883-025-04026-6, 39815189 PMC11734401

[31] Sousa JA Pancorbo O Simonetti R Llull L Coscojuela P Blasco J . Influence of spot sign on the association between rapidly achieving blood pressure reduction and intracerebral haemorrhage outcomes. Eur Stroke J. (2026) 11:aakaf024. doi: 10.1093/esj/aakaf024, 41614513 PMC12866633

[32] Seiffge DJ Fandler-Höfler S Du Y Goeldlin MB Jolink WMT Klijn CJM . Intracerebral haemorrhage - mechanisms, diagnosis and prospects for treatment and prevention. Nat Rev Neurol. (2024) 20:708–23. doi: 10.1038/s41582-024-01035-w, 39548285

[33] Hainsworth AH Markus HS Schneider JA. Cerebral small vessel disease, hypertension, and vascular contributions to cognitive impairment and dementia. Hypertension. (2024) 81:75–86. doi: 10.1161/HYPERTENSIONAHA.123.19943, 38044814 PMC10734789

[34] Charidimou A Boulouis G Frosch MP Baron J-C Pasi M Albucher JF . The Boston criteria version 2.0 for cerebral amyloid angiopathy: a multicentre, retrospective, MRI-neuropathology diagnostic accuracy study. Lancet Neurol. (2022) 21:714–25. doi: 10.1016/S1474-4422(22)00208-3, 35841910 PMC9389452

[35] Ridha M Hannawi Y Murthy S Carvalho Poyraz F Kumar A Park S . Premorbid blood pressure control modifies risk of DWI lesions with acute blood pressure reduction in intracerebral hemorrhage. Hypertension. (2024) 81:2113–23. doi: 10.1161/HYPERTENSIONAHA.124.23271, 39069917 PMC11410531

[36] Butcher KS Buck B Dowlatshahi D Gioia LC Kate M Klahr AC . Acute blood pressure lowering and risk of ischemic lesions on MRI after intracerebral hemorrhage. JAMA Neurol. (2025) 82:543–50. doi: 10.1001/jamaneurol.2025.0586, 40257759 PMC12012699

[37] Clocchiatti-Tuozzo S Qureshi AI Rivier CA Huo S Matouk C Payabvash S . Functional outcomes after intensive blood pressure reduction in deep and lobar intracerebral hemorrhage. Neurology. (2026) 106:e214860. doi: 10.1212/WNL.0000000000214860, 41962119

[38] Kang K Shi K Liu J Li N Wu J Zhao X. Autonomic dysfunction and treatment strategies in intracerebral hemorrhage. CNS Neurosci Ther. (2024) 30:e14544. doi: 10.1111/cns.14544, 38372446 PMC10875714

[39] Wagstaff H Ledyard HK. Blood pressure Management in Neurologic Emergencies. Emerg Med Clin North Am. (2026) 44:123–41. doi: 10.1016/j.emc.2025.08.008, 41260848 PMC12768389

[40] Zeng W-Z Marshall KL Min S Daou I Chapleau MW Abboud FM . PIEZOs mediate neuronal sensing of blood pressure and the baroreceptor reflex. Science. (2018) 362:464–7. doi: 10.1126/science.aau6324, 30361375 PMC6563913

[41] Magid-Bernstein J Girard R Polster S Srinath A Romanos S Awad IA . Cerebral hemorrhage: pathophysiology, treatment, and future directions. Circ Res. (2022) 130:1204–29. doi: 10.1161/CIRCRESAHA.121.319949, 35420918 PMC10032582

[42] Chen Y Chen S Chang J Wei J Feng M Wang R. Perihematomal edema after intracerebral hemorrhage: an update on pathogenesis, risk factors, and therapeutic advances. Front Immunol. (2021) 12:740632. doi: 10.3389/fimmu.2021.740632, 34737745 PMC8560684

[43] Ramanujam RK Lavi Y Poole LG Bassani JL Tutwiler V. Understanding blood clot mechanical stability: the role of factor XIIIa-mediated fibrin crosslinking in rupture resistance. Res Pract Thromb Haemost. (2025) 9:102871. doi: 10.1016/j.rpth.2025.102871, 40496848 PMC12150072

[44] Ramanujam RK Garyfallogiannis K Litvinov RI Bassani JL Weisel JW Purohit PK . Mechanics and microstructure of blood plasma clots in shear driven rupture. Soft Matter. (2024) 20:4184–96. doi: 10.1039/d4sm00042k, 38686609 PMC11135145

[45] Zakharov A Awan M Gopinath A Lee S-JJ Ramasubramanian AK Dasbiswas K. Clots reveal anomalous elastic behavior of fiber networks. Sci Adv. (2024) 10:eadh1265. doi: 10.1126/sciadv.adh1265, 38198546 PMC10780871

[46] Kliuchnikov E Dagklis AG Litvinov RI Marx KA Weisel JW Bassani JL . Strength, deformability, damage and fracture toughness of fibrous material networks: application to fibrin clots. Acta Biomater. (2025) 201:347–59. doi: 10.1016/j.actbio.2025.05.057, 40414265

[47] Wang L Luo Y Yuan Q Xiong X Jin G Dong J . Impact of FXIII levels on hematoma expansion and outcomes in patients with spontaneous intracranial hemorrhage: a prospective observational cohort study of 132 patients. Neurocrit Care. (2026) 45:581–92. doi: 10.1007/s12028-026-02513-3, 41933247 PMC13642390

[48] Liu S Bahmani A Ghezelbash F Li J. Fibrin clot fracture under cyclic fatigue and variable rate loading. Acta Biomater. (2024) 177:265–77. doi: 10.1016/j.actbio.2024.01.046, 38336270

[49] Liu D Jia Z Liu B Hou J Bao R Wang M. Molecular creep induced fatigue rupture of fibrin clots. Adv Sci (Weinh). (2025) 12:e05109. doi: 10.1002/advs.202505109, 40776554 PMC12520524

[50] Jin T Fei M Luo S Wang H. Piezo1 as a potential player in intracranial hemorrhage: from perspectives on biomechanics and hematoma metabolism. J Biomed Res. (2024) 38:436–47. doi: 10.7555/JBR.37.20230241, 38808569 PMC11461532

[51] Zhao H Zhang K Tang R Meng H Zou Y Wu P . TRPV4 blockade preserves the blood-brain barrier by inhibiting stress Fiber formation in a rat model of intracerebral hemorrhage. Front Mol Neurosci. (2018) 11:97. doi: 10.3389/fnmol.2018.00097, 29636662 PMC5880899

[52] Du C Wu Y Geng Y Ali A Li Z Liu Z . Glibenclamide attenuates brain edema after intracerebral hemorrhage through preservation of blood-brain barrier integrity and Glymphatic function. Neurochem Res. (2026) 51:103. doi: 10.1007/s11064-026-04723-9, 41806094

[53] Zhao J Song C Li D Yang X Yu L Wang K . Efficacy and safety of glibenclamide therapy after intracerebral haemorrhage (GATE-ICH): a multicentre, prospective, randomised, controlled, open-label, blinded-endpoint, phase 2 clinical trial. EClinicalMedicine. (2022) 53:101666. doi: 10.1016/j.eclinm.2022.101666, 36177443 PMC9513728

[54] Chen X Wang L Wang N Li C Hang H Wu G . An apolipoprotein E receptor mimetic peptide decreases blood-brain barrier permeability following intracerebral hemorrhage by inhibiting the CypA/MMP-9 signaling pathway via LRP1 activation. Int Immunopharmacol. (2024) 143:113007. doi: 10.1016/j.intimp.2024.113007, 39486173

[55] Guan X Li M Li H Guo Z Ullah MS Liu X . The role of MMPs in intracerebral hemorrhage. Front Cell Dev Biol. (2025) 13:1667228. doi: 10.3389/fcell.2025.1667228, 41480312 PMC12754015

[56] Tang J Yue J Tao Y Zhao G Yi X Zhang M . Neutrophil extracellular traps induce brain edema around intracerebral hematoma via ERK-mediated regulation of MMP9 and AQP4. Transl Stroke Res. (2025) 16:1461–73. doi: 10.1007/s12975-024-01318-w, 39733198 PMC12391242

[57] Alluri H Grimsley M Anasooya Shaji C Varghese KP Zhang SL Peddaboina C . Attenuation of blood-brain barrier breakdown and Hyperpermeability by Calpain inhibition. J Biol Chem. (2016) 291:26958–69. doi: 10.1074/jbc.M116.735365, 27875293 PMC5207131

[58] Rasmussen MK Mestre H Nedergaard M. Fluid transport in the brain. Physiol Rev. (2022) 102:1025–151. doi: 10.1152/physrev.00031.2020, 33949874 PMC8897154

[59] Chen W Liang C Peng S Bao S Xue F Lian X . Aquaporin-4 activation facilitates glymphatic system function and hematoma clearance post-intracerebral hemorrhage. Glia. (2025) 73:368–80. doi: 10.1002/glia.24639, 39530196 PMC11662979

[60] Puy L Mazzoleni V Samarasekera N Morotti A Tsai H-H Gaberel T . Hematoma resorption in intracerebral hemorrhage: a translational overview on pathophysiology, biomarkers, and innovative therapies. Transl Stroke Res. (2025) 16:2350–63. doi: 10.1007/s12975-025-01389-3, 41075069

[61] Mukherjee U Chowdhury S Nagada KK Hasan MM Ghosh B Joshi A . Glymphatic system and intracerebral hemorrhage: identifying molecular targets for future therapeutic advancements. Ageing Res Rev. (2025) 112:102900. doi: 10.1016/j.arr.2025.102900, 40998175

[62] Sandset EC Palaiodimou L Jahr SH Ho L Fischer U Katsanos AH . 2025 Update to European stroke organisation (ESO) guideline on blood pressure management in acute ischaemic stroke and intracerebral haemorrhage. Eur Stroke J. (2026) 11:aakag004. doi: 10.1093/esj/aakag004 PMC13151662 42095756

[63] Puissant M Ganti L. Acute management of spontaneous intracerebral hemorrhage (ICH) in the emergency department. Int J Emerg Med. (2026) 19:35. doi: 10.1186/s12245-026-01126-z, 41618143 PMC12857004

[64] Chen Y Zhao G Xia X. Acute kidney injury after intracerebral hemorrhage: a mini review. Front Med (Lausanne). (2024) 11:1422081. doi: 10.3389/fmed.2024.1422081, 38988361 PMC11233433

[65] Parry-Jones AR Sammut-Powell C Paroutoglou K Birleson E Rowland J Lee S . An intracerebral hemorrhage care bundle is associated with lower case fatality. Ann Neurol. (2019) 86:495–503. doi: 10.1002/ana.25546, 31291031 PMC6771716

[66] Ma L Hu X Song L Chen X Ouyang M Billot L . The third intensive care bundle with blood pressure reduction in acute cerebral Haemorrhage trial (INTERACT3): an international, stepped wedge cluster randomised controlled trial. Lancet. (2023) 402:27–40. doi: 10.1016/S0140-6736(23)00806-1, 37245517 PMC10401723

[67] Khan M Ouyang M Wasay M Ma L Hu X Chen X . Clinical characteristics and outcomes of intracerebral haemorrhage in young vs older adults: insights from the INTERACT3 trial. Eur Stroke J. (2026) 11:aakag040. doi: 10.1093/esj/aakag040, 42378499 PMC13317946

[68] Zhao X Zhang W Zhang Y Liu X-A. Gastrointestinal dysfunction after brain injury: mechanisms and the role of the brain-gut axis. World J Gastroenterol. (2026) 32:115731. doi: 10.3748/wjg.v32.i10.115731, 41809453 PMC12968606

[69] Wen X Dong H Zou W. The role of gut microorganisms and metabolites in intracerebral hemorrhagic stroke: a comprehensive review. Front Neurosci. (2024) 18:1346184. doi: 10.3389/fnins.2024.1346184, 38449739 PMC10915040

[70] Sun Y Zhou D Liu A Zhou Y Zhao Y Yuan Y . Liangxue Tongyu prescription exerts neuroprotection by regulating the microbiota-gut-brain axis of rats with acute intracerebral hemorrhage. Brain Res Bull. (2025) 220:111186. doi: 10.1016/j.brainresbull.2024.111186, 39746523

[71] Jiang H Zeng W Zhu F Zhang X Cao D Peng A . Exploring the associations of gut microbiota with inflammatory and the early hematoma expansion in intracerebral hemorrhage: from change to potential therapeutic objectives. Front Cell Infect Microbiol. (2025) 15:1462562. doi: 10.3389/fcimb.2025.1462562, 39963412 PMC11830820

[72] Tao W-Q Fang H-Y Zou Z-Q Luo Y Li Y-F. Impacts of acupuncture on blood pressure and hematoma in patients of cerebral hemorrhage at the early stage. Zhongguo Zhen Jiu. (2014) 34:426–30. 25022108

[73] Liu S Wang Z Su Y Qi L Yang W Fu M . A neuroanatomical basis for electroacupuncture to drive the vagal-adrenal axis. Nature. (2021) 598:641–5. doi: 10.1038/s41586-021-04001-4, 34646018 PMC9178665

[74] Zeng L Yan J. Acupuncture at Zusanli (ST36) for the benefits of sepsis-laboratory evidence for “preventive treatment of disease” and “holistic concept” in the protective effects of acupuncture: a literature review of rodent studies. Front Neurol. (2025) 16:1645906. doi: 10.3389/fneur.2025.1645906, 41059507 PMC12499358

